# Anatomy of male and female genitalia of *Acanthoscelidesobtectus* (Say, 1831) (Coleoptera, Chrysomelidae, Bruchinae) in interaction

**DOI:** 10.3897/zookeys.1177.101621

**Published:** 2023-08-30

**Authors:** Michael Schmitt, Aileen Neumann, Shou-Wang Lin

**Affiliations:** 1 Universität Greifswald, Allgemeine & Systematische Zoologie, Loitzer Str. 26, 17489 Greifswald, Germany Universität Greifswald Greifswald Germany

**Keywords:** Bursa copulatrix, endophallus, female kicking, morphology

## Abstract

Armatures of the male intromittent copulatory structures have been surmised to increase male fitness by imposing physiological costs on female re-mating. Female kicking could, consequently, be a counterstrategy to avoid wounding or to prevent males from mating. The membranous endophallus of male *Acanthoscelidesobtectus* (Say, 1831) is armed with denticles. Checking if these denticles penetrate the wall of the female genital tract during copulation revealed that only the tip of the median lobe of the aedeagus is intromitted into the female genital opening during copulation. The everted endophallus extends over the full length of the ovipositor, and the spermatophore is placed in the bursa. Identification by means of light microscopy and Micro-CT of the exact relative position of male and female copulatory organs while mated confirmed that the denticles do not cause wounds in the vagina wall. Parts of the inner wall of the bursa copulatrix are covered with inward pointing denticles. Already mated females kick mounting males by vehement movements of their hind legs, thereby preventing mating. In contrast, virgin females usually accept the first male they encounter and terminate copulation by slower movements of their hind legs. The same applied to females who accepted re-mating the second day after the first copulation. *Acanthoscelidesobtectus* females kick males off to prevent rather than to terminate copulation. Copulatory structures as well as behaviour may have different functional roles in different beetle species, even within the Bruchinae.

## Introduction

The evolutionary interests of males and females regularly differ due to the different amounts of resources invested in reproduction. There is also a high differential in certainty of parentage between males and females. This leads to sexual conflict, and this conflict resulted in evolutionarily frequent morphological and behavioural adaptations in males to induce wounds in females during copulation and respective counteradaptations in females (Parker 1979; Arnqvist and Rowe 2005). In many insect taxa the male intromittent organ is armed with hooks, spines or denticles (Rönn and Hotzy 2012). This fact had been observed in seed beetles (Bruchinae) long before a possible functional explanation was hypothesised, see, e.g., the drawings of aedeagi in Borowiec (1987), Johnson (1990), or Schmitt (1985: fig. 40). When Crudgington and Siva-Jothy (2000) found that the spines of the everted endophallus of male cowpea weevils, *Callosobruchusmaculatus* (Fabricius, 1775) (Chrysomelidae: Bruchinae), perforated the wall of the bursa copulatrix in the female, they conjectured that this kind of genital damage prevents females from re-mating thereby increasing the fitness of the male by helping the male to control copulation duration or by decreasing the probability of subsequent matings of the female with other males. However, Edvardsson and Tregenza (2005) found no reluctance to re-mate in female *C.maculatus*, and Rönn and Hotzy (2012) showed that the male spines probably do not function as an anchor that prevents the male being kicked off, a possible alternative functional role suggested, e.g., by Edvardsson and Tregenza (2005). Female *C.maculatus* regularly kick off males during mating and by doing so terminate copulation and presumably reduce the probability of wounding (van Lieshout et al. 2014). Dougherty and Simmons (2017) studied *C.maculatus* pairs in copula by means of X-ray micro-CT scanning and found a temporal separation between the onset of wounding and the onset of female kicking.

**Figure 1.**
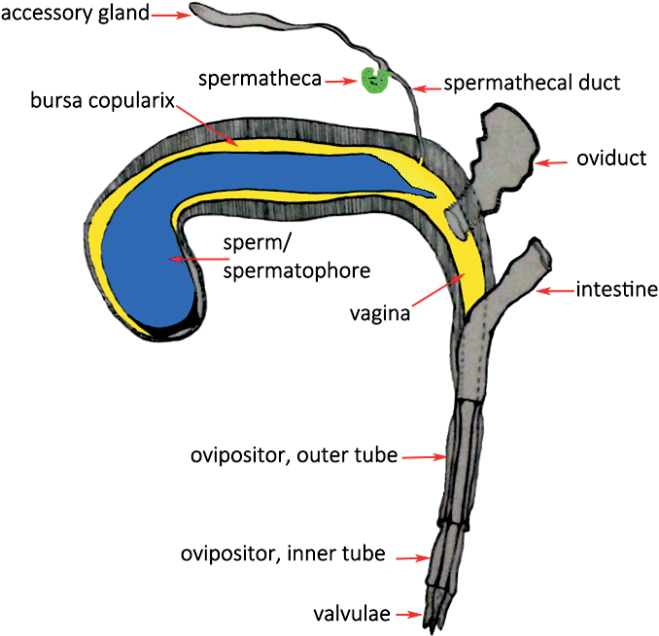
The female genital tract of *A.obtectus*. Schematic drawing after Huignard (1968).

Female bean weevils, *Acanthoscelidesobtectus* (Say, 1831), have an ovipositor consisting of an internal and an external sclerotised tube through which the membranous vagina extends (Fig. 1). The vagina is proximally enlarged and forms a blind ending, the bursa copulatrix. There the spermatophore is placed during copulation. The spermathecal duct reaches the genital tract at the transition between bursa and vagina near the opening of the oviduct (Huignard 1968).

**Figure 2.**
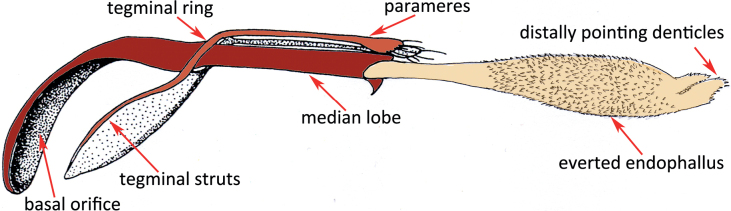
The male copulatory organ of *A.obtectus*. Schematic drawing after Schmitt (1985) and Düngelhoef and Schmitt (2006).

The male copulatory organ, the aedeagus, consists of a sclerotised median lobe, essentially a tube through which the ejaculatory duct runs from the basal orifice to the distal opening, and the tegmen that forms a ring around the median lobe and extends basically into paired struts and distally into paired parameres. The ejaculatory duct is distally enlarged and forms a membranous inflatable enlargement, the endophallus (Fig. 2).

Since the endophallus of male bean weevils is equipped with denticles or spicules (Düngelhoef and Schmitt 2006), we wanted to know if these denticles could also perforate the wall of the bursa copulatrix like the spines in *C.maculatus*. To this aim, we studied the anatomy of mated pairs with light microscopy and micro-CT. We also conducted mating experiments to explore female mating behaviour depending on their reproductive status.

Cowpea weevils and bean weevils are cosmopolitan pests on stored products. Therefore, the life history of these two species has been studied for a long time and is well known (e.g., Zacher 1933; Devi and Devi 2014). Since their larvae develop in dry legume seeds, these beetles can easily be kept in the laboratory.

## Materials and methods

### Animal keeping

A live population of *Acanthoscelidesobtectus* beetles (bean weevils) that we obtained from Dr. Thomas Degenkolb, Justus-Liebig-Universität Gießen, Germany, was kept at room temperature in the lab building of the Zoological Institute of the University of Greifswald, Germany, in a transparent plastic container of 23 × 14 × 15 cm (L × W × H) with a close-mesh fabric covered airing opening at room temperature of ca. 21 °C. They fed on and developed in organic bean seeds of ca. 1 cm length.

### Light microscopy

Ten females and males were randomly taken from the breeding container and set in a block bowl of 4 × 4 cm. When they copulated they were fixed by liquid nitrogen and dissected in distilled water or 96% ethanol under an Olympus Stereomicroscope SZ4045. The isolated genitalia were studied using an Olympus CX40RF200 or an Olympus BX60 equipped with a Zeiss AxioVision 4.8 digital camera. We used a manually sharpened minutien pin to dissect the isolated coupled male and female genitalia that were glued onto a surface with a viscose Polyvinylpyrrolidone solution. To trace the progress of sperm ingression into the bursa copulatrix we fixed five pairs 3, 5, 6, 7, and 8 minutes after the start of the copulation.

### Micro-CT

Two copulating pairs were fixed with liquid nitrogen, transferred into 99% methylated spirit, and stored at -41 °C. The probes were contrasted in 99% ethanol and 1% Iodine. They were critical-point-dried in a Leica EM CPD300 and mounted on a metal pole of 40 × 1.8 mm. Using an Xradia MicroXCT-200 (Carl Zeiss X-ray Microscopy Inc.) one pair was scanned at 10× magnification at 40 kV and 8 W and a pixel size of 1.15 µm, the other at 40 kV at 6 W and a pixel size of 2.22 µm, yielding 980 virtual sections for each pair. We analysed the data and reconstructed the 3D picture by means of Amira 5.6.0 (FEI Visualization Science Group, Burlington, USA).

### Mating tests

Virgin beetles hatched from singly kept bean seeds were sexed and set into a block bowl of 4 × 4 cm together with a randomly chosen male. Thirty-three tests were performed. Re-mating trials were done with 14 females of these on day 1 after the first copulation and with 23 females on day 2. In the re-mating trials, the females were offered up to three different males for 10 min each.

## Results

### Light microscopy

**Figure 3.**
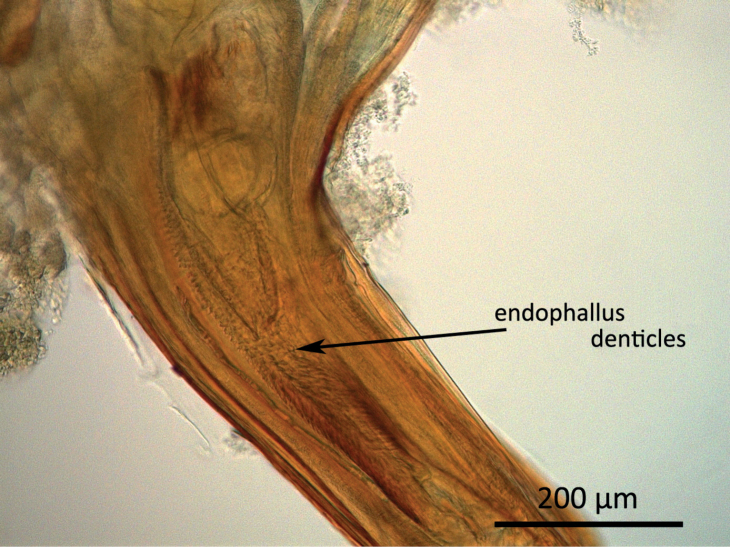
Light microscopic image of a dissected *A.obtectus* specimen. Proximal section of the genital tract of a mated female with endophallus inside.

Dissecting the genitalia of mated been weevil pairs revealed the position of the endophallus inside the female genital tract. Fig. 3 shows the proximal section of the ovipositor with the inner tube in repose. The endophallus spines can clearly be seen, however no traces of perforations of the vagina wall were apparent. The denticles on the tip of the endophallus are longer than those on the main part and point distally.

**Figure 4.**
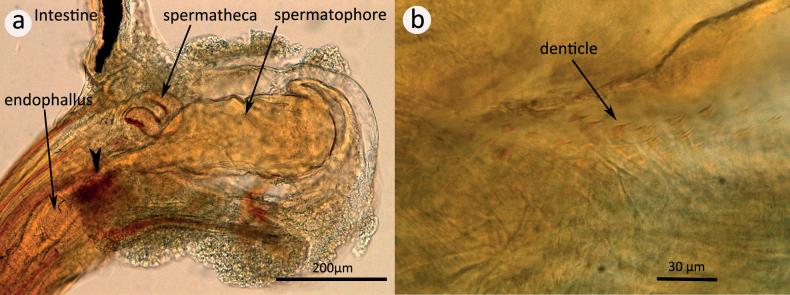
Dissected bursa copulatrix of a mated *A.obtectus* female **a** relative position of endophallus and spermatophore inside the bursa. The arrowhead points to the tip of the endophallus **b** detail showing the inwards pointing denticles on the inner wall of the bursa.

The tip of the endophallus reaches the transition of the vagina into the bursa (arrowhead in Fig. 4a). We found that parts of the inner wall of the bursa are covered with fine denticles (Fig. 4b). These denticles point towards the proximal end of the bursa.

### Micro-CT

**Figure 5.**
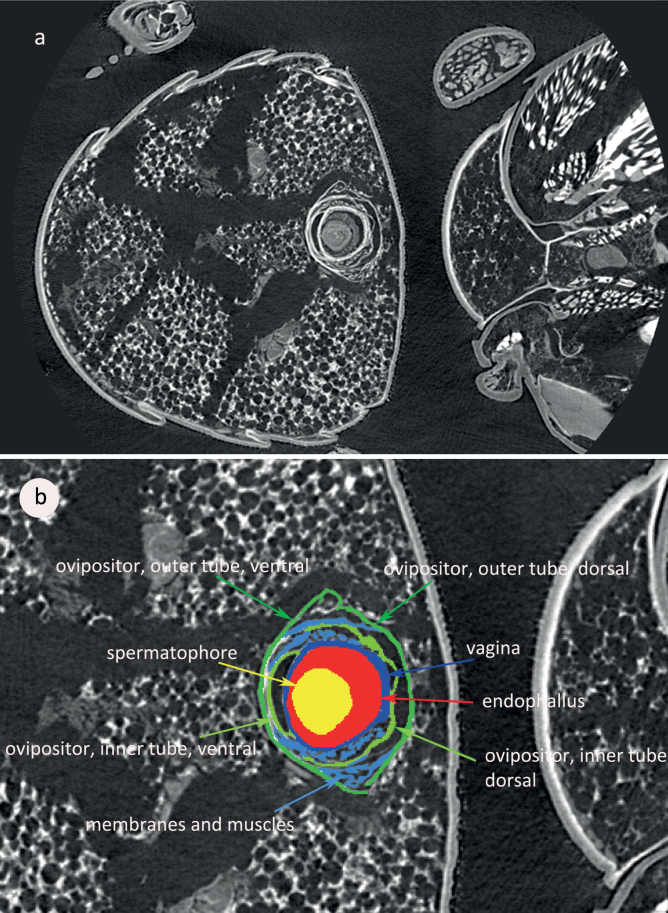
A virtual section through the abdomens of male (right) and female (left) *A.obtectus* fixed in copula. Micro-CT photograph **a** unaltered virtual slice, pixel size 1.15 µm **b** elements of the copulatory organs and the spermatophore labelled (“segmented”) in different colours. Red and yellow: male structures, green and blue: female structures.

Analysis of the micro-CT virtual sections revealed that the inner and the outer tube of the ovipositor are made up of two half-tubes each, a dorsal and a ventral one. Outer and inner tube of the ovipositor are connected by membranes and muscles that allow for extension and retraction of the tubes (Fig. 5).

**Figure 6.**
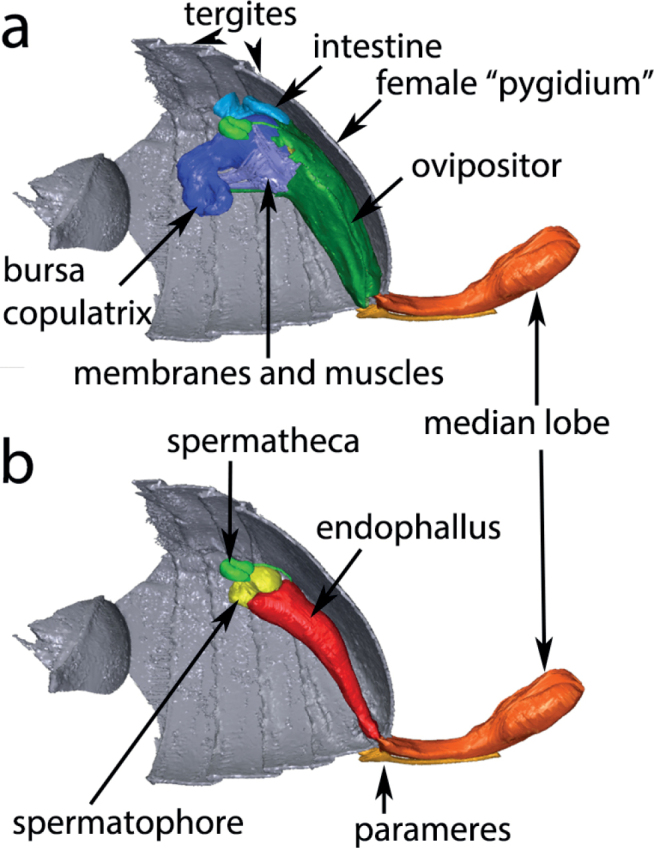
Micro-CT photograph of the 3D reconstruction of the coupled male and female *A.obtectus* genitalia, fixed during copulation. The spermatophore had not yet completely filled the bursa copulatrix at the time of fixation **a** the outer tube of the ovipositor covers the inner tube and the vagina **b** all components of the female genital tract except the spermatheca removed to show the shape and the extension of the everted endophallus.

The endophallus carrying the spermatophore lies inside the vagina that stretches through the inner tube of the ovipositor. Only the tip of the median lobe of the aedeagus is inserted into the female genital opening during copulation, while the parameres remain outside the female abdomen (Fig. 6). The everted endophallus extends through the vagina over the whole length of the retracted ovipositor to the entrance of the bursa copulatrix (Fig. 6b). The female genital tract lies, at least during copulation, immediately under the last visible tergite, the so-called pygidium.

### Mating tests

Of the 33 virgin females, 22 (73%) accepted copulations without kicking or wriggling, and two after initial kicking. Copulations of these 24 females ended not by the females kicking off the males but either the females pushed the males away by slow hind leg movements, wriggled their body, or simply ran away after the male had dismounted. Copulation lasted between 6:00 and 11:35 minutes, on average 9:24 minutes. Of the nine females who did not mate, five prevented mating by kicking the males away and three moved away. In one case the female seemed to accept a male but the male did not successfully mate.

All females that were tested for re-mating on day 1 after the first copulation (*n* = 14) prevented a second copulation by kicking off a male that aimed at mounting. Eight of the 23 females that were tested on the second day after their first copulation, accepted re-mating. Copulations lasted between 5:35 and 11:37 minutes. The 15 that did not mate kicked the male or ran away.

## Discussion

The central question of our study could be answered: in *Acanthoscelidesobtectus*, the denticles on the surface of the endophallus do not perforate the wall of the vagina during copulation. The function of the denticles on the endophallus might be to enhance the friction between male and female copulatory organs when the endophallus is inflated inside the female genital tube. Kingsolver (1970) surmised that in seed beetles these armatures provide a certain foothold of the male during copulation, but it is unclear if they have an impact on the copulation duration. Since the spikes on the tip of the endophallus point distally (Fig. 2), they might also push the spermatophore into the bursa copulatrix. These distally pointing denticles could as well pierce the spermatophore and by doing so aid sperm release, as it was suggested for *Callosobruchusmaculatus* by Dougherty and Simmons (2017).

An alternative functional role could be the mechanical stimulation of the female during copulation (see Eberhard 1985: 157–166). Simmons (2014) summarises that “non-intromittent genitalia are subject to sexual selection through their effects on mating success, while intromittent genitalia are subject to selection through their effects on fertilisation success”. This underlines the idea that the endophallus ornaments enhance male fitness by stimulating the female and so possibly signalling male quality, i.e., prospective fitness.

The male inserts only the tip of the median lobe into the female genital opening (Düngelhoef and Schmitt 2006) and the parameres remain outside of the female body and function most probably as “genital feelers” (Düngelhoef and Schmitt 2010). The sclerotised parts of the copulatory organs of both sexes do not couple mechanically, i.e., there is no sign of a “lock-and-key” mechanism. Thus, our observations are in accordance with the general hypothesis of Eberhard (1985) that the copulatory organs are shaped during evolution by sexual selection. Other than many Coccinellidae (Yadav and Pervez 2022), males of *Acanthoscelidesobtectus* do not perform vigorous shaking during copulation. Consequently, there is no external sign of movements of the male genitalia inside the female genital tract. The males, however, move the tips of the parameres softly over the last sternite of the females during mating (Düngelhoef and Schmitt 2006). These movements are governed by muscles that are in direct connection to the muscles everting the endophallus. This suggests a coupling of movements of the parameres and of the endophallus so that a stimulatory function is easily possible.

The inner tube of the ovipositor is in repose slipped into the outer one “like a telescope” (Lindroth and Palmén 1970), similarly to the Eumolpinae (Flowers and Eberhard 2006). The virtual cross-section through the abdomen of a female in copula (see Fig. 5) shows that the ovipositor tubes are each composed of two half-tubes. These half-tubes are most probably phylogenetically and ontogenetically derivatives of the tergites and sternites of the female 8^th^ and 9^th^ abdominal segments (Verhoeff 1893). The denticles on some areas of the wall of the bursa copulatrix can possibly keep a spermatophore in place and prevent it from sliding out.

While *Callosobruchusmaculatus* virgin and mated females regularly terminated copulation by kicking off the mating male (van Lieshout et al. 2014), we found that *A.obtectus* mated females prevented subsequent mating by kicking off males. When the males did not terminate the copulation by dismounting, the females terminated the copulation by wriggling their body and/or pushing the males with their hind legs. It seems that female kicking plays a different role in the two species. Seemingly, *C.maculatus* females kick to terminate copulation while *A.obtectus* females kick to prevent copulation. However, Mbata et al. (1997) observed that mated females of *Callosobruchussubinnotatus* (Pic, 1914) in some cases prevented males from mating by kicking them off. Thus, female kicking to prevent mating is either a species-specific behaviour or *A.obtectus* females can also terminate mating by kicking males off but did not so in our trials, for whatever reasons.

Earlier authors have found cuticular spicules (small needle-like processes) or denticles (small tooth-like sclerotised structures) on the endophallus (or “internal sac”) in all investigated seed beetle species (e.g., Hoffmann 1945; Borowiec 1987). Düngelhoef and Schmitt (2010) found endophallus denticles in *Mecynoderacoxalgica* (Boisduval, 1835) of the chrysomelid subfamily Sagrinae (the putative sister group of the Bruchinae, Reid 2014). We hypothesise that these structures were present in the ancestor of Bruchinae and Sagrinae. No such armatures were found in a reed beetle (Donaciinae) and a shining leaf beetle (Criocerinae) (Schmitt and Uhl 2015). Flowers and Eberhard (2006) described microspicules, hooks, spines, and needles on the endophalli of Neotropical Eumolpinae and Galerucinae. Most probably such structures are phylogenetically as old as the earliest Coleoptera and were reduced and/or modified many times independently.

In the groups in which spines or denticles occur on the endophallus, they are of different length, shape, and position in the different species where they were observed. This suggests that these structures fulfil different functional roles in different groups, e.g., terminating copulation in *Callosobruchus* species or preventing copulation in *A.obtectus*. Van Haren et al. (2017) found that ablating genital armatures in *Callosobruchussubinnotatus* (Pic, 1914) males resulted in a reduction in female egg production. This means that post-mating sexual selection might play a crucial part in the evolution of the equipment of male genitalia with denticles, hooks, or spines. As Flowers and Eberhard (2006) have stated, the morphological diversity of leaf beetle genitalia certainly also represents a diversity of functional roles.

## Additional information

### Author ORCIDs

Michael Schmitt https://orcid.org/0000-0001-7377-3643

### Data availability

All of the data that support the findings of this study are available in the main text or Supplementary Information.
